# The Shock Pulse Index and Its Application in the Fault Diagnosis of Rolling Element Bearings

**DOI:** 10.3390/s17030535

**Published:** 2017-03-08

**Authors:** Peng Sun, Yuhe Liao, Jin Lin

**Affiliations:** 1 Shaanxi Key Laboratory of Mechanical Product Quality Assurance and Diagnostics, Xi’an Jiaotong University, Xi’an 710049, China; tracysun@stu.xjtu.edu.cn; 2 State Key Laboratory for Manufacturing Systems Engineering, Xi’an Jiaotong University, Xi’an 710049, China; jinglin@mail.xjtu.edu.cn

**Keywords:** fault diagnosis, shock pulse index, maximum correlated kurtosis deconvolution, teager energy operator, rolling element bearings

## Abstract

The properties of the time domain parameters of vibration signals have been extensively studied for the fault diagnosis of rolling element bearings (REBs). Parameters like kurtosis and Envelope Harmonic-to-Noise Ratio are the most widely applied in this field and some important progress has been made. However, since only one-sided information is contained in these parameters, problems still exist in practice when the signals collected are of complicated structure and/or contaminated by strong background noises. A new parameter, named Shock Pulse Index (SPI), is proposed in this paper. It integrates the mutual advantages of both the parameters mentioned above and can help effectively identify fault-related impulse components under conditions of interference of strong background noises, unrelated harmonic components and random impulses. The SPI optimizes the parameters of Maximum Correlated Kurtosis Deconvolution (MCKD), which is used to filter the signals under consideration. Finally, the transient information of interest contained in the filtered signal can be highlighted through demodulation with the Teager Energy Operator (TEO). Fault-related impulse components can therefore be extracted accurately. Simulations show the SPI can correctly indicate the fault impulses under the influence of strong background noises, other harmonic components and aperiodic impulse and experiment analyses verify the effectiveness and correctness of the proposed method.

## 1. Introduction

The potential failure of rotating machinery has a close relationship with the safe operation of industrial production, so a powerful fault diagnosis technology is the essential security component of industrial production. Generally, fault diagnosis methods can be categorized into model-based methods, signal-based methods, knowledge-based methods, hybrid methods (methods that combine at least two methods), and active fault diagnosis methods [1,2]. Due to the fact they do not require an explicit or complete model, signal-based methods are particularly suitable for monitoring and diagnosis for complex industrial processes where explicit system models are unavailable or challenging to derive [2]. The signal-based methods can be classified into time-domain [3], frequency-domain [4] and time-frequency approaches [5] according to the different features extracted from the signals [1].

Rolling element bearings (REBs), as an important component of rotating machinery, are widely used and the running health condition of REBs is of great importance to the operating stability and safety of the related machinery. Therefore, study on the theory and method of the REB fault diagnosis has always been a hotspot in this research area. Many scholars and engineers around the world have made enormous efforts on this topic and great achievements have been achieved [6]. As to some of the vibration analysis based REB fault diagnosis methods, how to effectively extract the fault feature related impulse components from the vibration signal is always been the critical issue that needs to be solved first of all. Here the difficulty lies primarily in that, besides the fault feature frequency components, the collected vibration signal also contains some other interference components [6]. In some serious situations, especially at the early stage of the REB faults, useful information with comparatively low amplitude could even be totally submerged by strong background noises, so the fault information extraction from the raw vibration signal is an important part of fault diagnosis and condition monitoring of the REB and time-domain features and frequency-domain features are the first choice because of their simplicity and definite physical [7].

Considering the sensitivity to impact signal, which is the main characteristic of faulty REB vibrations, one of the time domain higher-order statistics, the kurtosis that is very sensitive for impact signal [8,9,10], is introduced here. It was first studied by Dyer et al. and they suggested that a kurtosis greater than 3 could indicate the emergence of bearing faults [11]. However, since they calculate the kurtosis directly with the noised vibration signal, the result could suffer interference from the background noise. In view of this, Dwyer then proposed the spectral kurtosis (SK), which can locate the impulse components in the frequency domain, to solve this problem [12]. Based on that, Antoni [13,14,15] further studied the theory of the SK and put forward the concept of fast kurtogram for optimal resonance frequency bands. Lei [16] introduced the wavelet packet transform (WPT), which was used as a filter to replace the FIR filters in kurtogram and the analysis accuracy was therefore improved. However, interference of strong non-Gaussian noise could still cause problems for the SK technique and lead to incorrect results [6,17]. Barszcz [17] then put forward a method named protrugram which is capable of detecting transients with much smaller signal-to-noise ratio than that of the SK-based fast kurtogram. The drawback of this method is that it needs prior knowledge of the signal, which hinders its application in practice. Xu [18,19] suggested a new ehnrgram based on Envelope Harmonic-to-Noise Ratio (EHNR). The advantage of the EHNR lies in that it is insensitive to the large aperiodic impulses and can efficiently extract the interested periodic impulses at the same time, but the EHNR could also get into trouble since it is very sensitive to the harmonic components, such as the rotating frequency component and its higher order harmonics, of the vibration signals.

However, there are some issues still worthy of discussion. Generally, besides the fault- related impulse components of interest, real collected vibration signals also contain some interference, such as background noises and rotating frequency-related harmonic components. In some cases, random impulses coming from the external environment further complicate the bearing fault diagnostic problem.

In view of that, a new parameter, named Shock Pulse Index (SPI), is proposed in this paper. It combines the mutual advantages of both the kurtosis and the EHNR at the same time and can therefore accurately help extract the periodic impulses from raw signals. Moreover, the SPI is insensitive to the aperiodic impulse and the rotating frequency-related harmonics. This in turn improves the robustness of the diagnostic process. Here background noise is a non-ignorable factor that should be carefully considered. Maximum Correlated Kurtosis Deconvolution (MCKD) [20] is an effective approach to highlight the fault-related periodic impulse components [21]. However, whether its parameter settings are proper or not has a direct influence on analysis results [22,23]. Therefore, a new way of parameter optimization for the MCKD based on the SPI is then implemented to ensure correct extraction of interested information. Finally, considering the modulation characteristics of the faulty bearing signal [6], a nonlinear differential operator, the Teager Energy Operator (TEO) [24,25], is used to estimate the energy required to find the Fault Characteristic Frequency (FCF). The amplitude envelope and instantaneous frequency of any modulated signal then can be obtained.

The paper is arranged as follows: the theory of this research is stated in Section 2. In Section 3, the properties of the SPI are illustrated by a simulation analysis. The method based on the SPI is proposed in Section 4. The performance of the novel method is illustrated by real data in Section 5. Problems in the research are discussed in Section 6. Finally, the conclusions are given in Section 7.

## 2. Theoretical Background

### 2.1. Teager Energy Opertor

For a signal x(t), the Teager energy operator Ψ is defined as [26,27,28]:

(1) $$\Psi \left[x\left(t\right)\right]={\left(\frac{\mathrm{dx}\left(t\right)}{\mathrm{dt}}\right)}^{2}-x\left(t\right)\left(\frac{d^{2}x\left(t\right)}{\mathrm{dt}}\right)={\left[\dot{x}\left(t\right)\right]}^{2}-x\left(t\right)\ddot{x}\left(t\right),$$

where $\dot{x}\left(t\right)$ and $\ddot{x}\left(t\right)$ are the first and the second derivative of x(t) with respect to time t, respectively. The energy operator in Equation (1) is defined for continuous time signals. Using difference to approximate derivatives, we replace t with ${\mathrm{nT}}_{s}$ ($T_{s}$ is the sampling period), x(t) with $x\left({\mathrm{nT}}_{s}\right)$ or simply x(n). Then:

(2) $$\dot{x}\left(t\right)=\left[x\left(n\right)-x\left(n-1\right)\right]/T_{s},$$

(3) $$\ddot{x}\left(t\right)=\left[x\left(n\right)-2x\left(n-1\right)+x\left(n-2\right)\right]/T_{s}^{2},$$

(4) $$\Psi \left[x\left(t\right)\right]=\left\{{\left[x\left(n-1\right)\right]}^{2}-x\left(n\right)x\left(n-2\right)\right\}\cdot T_{s}^{-2},$$

and ignoring the one-sample shift and the scaling by $T_{s}^{-2}$. Its counterpart for discrete time signals x(n) becomes:

(5) $$\Psi \left[x\left(n\right)\right]={\left[x\left(n\right)\right]}^{2}-x\left(n-1\right)x\left(n+1\right).$$

For a vibration displacement of the linear oscillator of undamped free vibration:

(6) x(t)=Acos(2πft+φ).

Its first and second derivatives, i.e., velocity and acceleration, are respectively:

(7) $$\dot{x}\left(t\right)=-2\pi \mathrm{Af}\sin\left(2\pi \mathrm{ft}+\varphi \right),$$

(8) $$\ddot{x}\left(t\right)=-4\pi^{2}{\mathrm{Af}}^{2}\cos\left(2\pi \mathrm{ft}+\varphi \right),$$

where A is the amplitude, f is the frequency, and φ is an initial phase.

Applying the Teager energy operator Ψ to the Equation (3) and substituting for its first and second derivatives by the $\dot{x}\left(t\right)$ and $\ddot{x}\left(t\right)$ from Equations (4) and (5), yields:

(9) $$\Psi \left[x\left(t\right)\right]={\left[\dot{x}\left(t\right)\right]}^{2}-x\left(t\right)\ddot{x}\left(t\right)=4\pi^{2}A^{2}f^{2}.$$

Further applying the Teager energy operator Ψ to the derivative of x(t), i.e., $\dot{x}\left(t\right)$, produces:

(10) $$\Psi \left[\dot{x}\left(t\right)\right]={\left[\ddot{x}\left(t\right)\right]}^{2}-\dot{x}\left(t\right)\dddot{x}\left(t\right)=16\pi^{4}A^{2}f^{4}.$$

The absolute amplitude and the frequency can then be obtained from Equations (6) and (7) as follows:

(11) $$\left|A\right|=\frac{\Psi \left[x\left(t\right)\right]}{\sqrt{\Psi \left[\dot{x}\left(t\right)\right]}},$$

(12) $$f=\frac{1}{2\pi }\sqrt{\frac{\Psi \left[\dot{x}\left(t\right)\right]}{\Psi \left[x\left(t\right)\right]}}.$$

This is what we called the TEO algorithm that can be generalized to signals with arbitrary time-varying amplitude and frequency. Thus the absolute value of instantaneous amplitude envelope a(t) and the instantaneous frequency f(t) can be estimated as:

(13) $$\left|a\left(t\right)\right|=\frac{\Psi \left[x\left(t\right)\right]}{\sqrt{\Psi \left[\dot{x}\left(t\right)\right]}},$$

(14) $$f\left(t\right)=\frac{1}{2\pi }\sqrt{\frac{\Psi \left[\dot{x}\left(t\right)\right]}{\Psi \left[x\left(t\right)\right]}}.$$

If no special instructions are provided, the envelope spectra of this paper are generated by the TEO.

### 2.2. Maximum Correlated Kurtosis Deconvolution

Inspired by the Minimum Entropy Deconvolution (MED) technique, an improved novel deconvolution technique named Maximum Correlated Kurtosis Deconvolution (MCKD) was proposed [20]. The MCKD technique is based on selecting a FIR filter to maximize the Correlated Kurtosis (CK), which takes advantage of the periodicity of the faults and requires no AR model stage prior to deconvolution, of the resulting signal which emphasizes high kurtosis while encouraging periodicity about a specific period.

Consider a discrete signal y(n) that is the response of the bearing excited by the fault impulses signal x(n). The essence of the MCKD algorithm is that searches for a FIR filter f(k) to maximize the CK of the signal x(n) recovered from the input signal:

(15) $$x\left(n\right)=\sum_{k=1}^{L}f\left(k\right)y\left(n-k+1\right),$$

where $f\left(k\right)={\left[f_{1}f_{2}\cdots f_{L}\right]}^{T}$, L is the length of the FIR filter.

The Correlated Kurtosis is defined as:

(16) $${\mathrm{CK}}_{M}\left(T\right)=\frac{\sum_{n=1}^{N}{\left(\prod_{m=0}^{M}x_{n-\mathrm{mT}}\right)}^{2}}{{\left(\sum_{n=1}^{N}x_{n}^{2}\right)}^{M+1}},$$

where T is the period of impulses and M is the shift number.

The optimization function of the MCKD is expressed as:

(17) $${\mathrm{MCKD}}_{M}\left(T\right)=\underset{f\left(k\right)}{\max}{\mathrm{CK}}_{M}\left(T\right)=\underset{f\left(k\right)}{\max}\frac{\sum_{n=1}^{N}{\left(\prod_{m=0}^{M}x_{n-\mathrm{mT}}\right)}^{2}}{{\left(\sum_{n=1}^{N}x_{n}^{2}\right)}^{M+1}}.$$

The above optimization problem is equivalent to solve Equation, as follows:

(18) $$\frac{d}{\mathrm{df}\left(k\right)}{\mathrm{CK}}_{M}\left(T\right)=0.$$

Solving the Equation (15) and the coefficients f(k) can be expressed by a matrix form as follows:

(19) $$f\left(k\right)=\frac{{∥\vec{x}∥}^{2}}{2{∥\vec{\beta }∥}^{2}}{\left(Y_{0}Y_{0}^{T}\right)}^{-1}\sum_{m=0}^{M}Y_{\mathrm{mT}}{\vec{\alpha }}_{m},$$

where ${\vec{\alpha }}_{m}=\left[\begin{array}{c} x_{1-\mathrm{mT}}^{-1}\left(x_{1}^{2}x_{1-T}^{2}\cdots x_{1-\mathrm{MT}}^{2}\right)\\ x_{2-\mathrm{mT}}^{-1}\left(x_{2}^{2}x_{2-T}^{2}\cdots x_{2-\mathrm{MT}}^{2}\right)\\ \vdots \\ x_{N-\mathrm{mT}}^{-1}\left(x_{N}^{2}x_{N-T}^{2}\cdots x_{N-\mathrm{MT}}^{2}\right) \end{array}\right]$, $\vec{\beta }=\left[\begin{array}{c} x_{1}x_{1-T}\cdots x_{1-\mathrm{MT}}\\ x_{2}x_{2-T}\cdots x_{2-\mathrm{MT}}\\ \vdots \\ x_{N}x_{N-T}\cdots x_{N-\mathrm{MT}} \end{array}\right]$, $Y_{\mathrm{mT}}={\left[\begin{array}{c} \begin{array}{ccc} y_{1-\mathrm{mT}} & y_{2-\mathrm{mT}} & \cdots \\ 0 & y_{1-\mathrm{mT}} & \cdots \\ \vdots & \vdots & \ddots \end{array}\\ 0\;0\;\cdots \end{array}\begin{array}{c} y_{N-\mathrm{mT}}\\ y_{N-1-\mathrm{mT}}\\ \begin{array}{c} \vdots \\ y_{N-L-\mathrm{mT}+1} \end{array} \end{array}\right]}_{L\times N}$.

Calculating the coefficients f(k) by Equation (16), the MCKD can extract the fault impulses from the vibration signal effectively.

### 2.3. Shock Pulse Index

#### 2.3.1. Kurtosis

Kurtosis as the fourth standardized moment can reflects the distribution characteristics of vibration signals and it was defined as:

(20) $$\mathrm{Kurtosis}\left(x\right)=\frac{\int_{-\infty }^{+\infty }{\left[x\left(t\right)-\bar{x}\right]}^{4}p\left(x\right)\mathrm{dx}}{\sigma^{4}},$$

where x(t) is the instantaneous of amplitude, $\bar{x}$ is the mean of amplitude, p(x) is the probability density and σ is the standard deviation.

**Figure 1:**
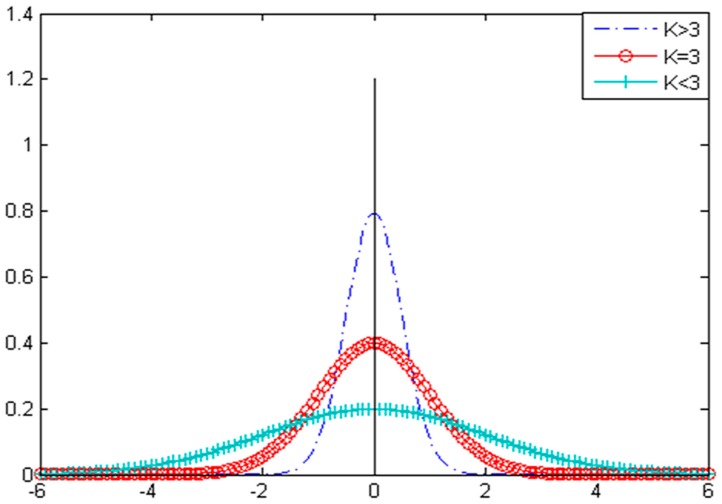
The outlier-proneness of a distribution.

As shown in Figure 1, kurtosis is a measure of how outlier-prone a distribution is. The kurtosis of the normal distribution is 3. Distributions that are more outlier-prone than the normal distribution have kurtosis greater than 3; distributions that are less outlier-prone have kurtosis less than 3. Therefore, kurtosis can effectively identify the impulse signal from the vibration signal.

#### 2.3.2. Envelope Harmonic-To-Noise Ratio

The harmonic-to-noise ratio (HNR) is an important characteristic parameter and it is used to describe the ratio of harmonic and noise components in speech signal analysis. In view of the fault-induced periodic impulses are usually modulated at resonance frequencies, the EHNR was proposed to eliminate the phenomenon of modulation [18,19]. The EHNR algorithm can be calculated as follows:

Obtain the envelope signal E[x(t)] by Hilbert transform of the measured signal x(t) and remove the DC component:

(21) $$x^{\prime }\left(t\right)=H\left\{x\left(t\right)\right\}=\frac{1}{\pi }\int_{-\infty }^{+\infty }\frac{x\left(\tau \right)}{t-\tau }d\tau ,$$

(22) $$E\left[x\left(t\right)\right]=\sqrt{x^{2}\left(t\right)+x^{\prime 2}\left(t\right)},$$

(23) E[x(t)]=E[x(t)]−mean{E[x(t)]}.

Computing the autocorrelation of E[x(t)]:

(24) $$r_{E\left[x\left(t\right)\right]}\left(\tau \right)=\int E\left[x\left(t\right)\right]E\left[x\left(t+\tau \right)\right]\mathrm{dt}.$$

**Figure 2:**
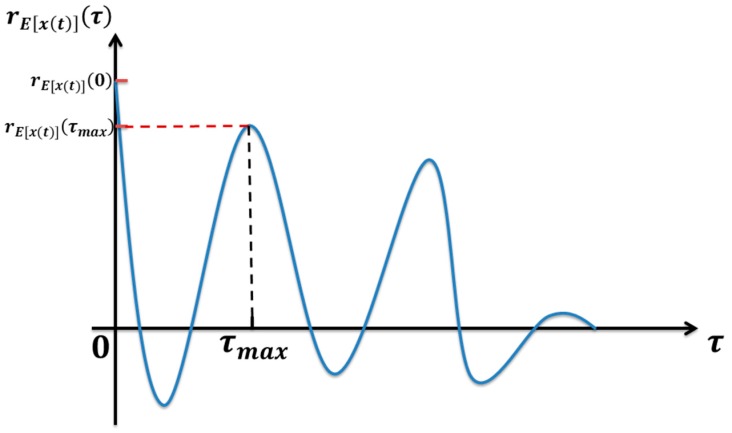
The autocorrelation function of the $r_{E\left[x\left(t\right)\right]}\left(\tau \right)$.

Then the EHNR can be represented as:

(25) $$\mathrm{EHNR}=\frac{r_{E\left[x\left(t\right)\right]}\left(\tau_{\max}\right)}{r_{E\left[x\left(t\right)\right]}\left(0\right)-r_{E\left[x\left(t\right)\right]}\left(\tau_{\max}\right)},$$

where τ is delay time, $\tau_{\max}$ is the delay time of the maximum of autocorrelation function in addition to the zero point, the point indicated in Figure 2, $x^{\prime }\left(t\right)$ is the Hilbert transform of the x(t), E[x(t)] is the amplitude of the Hilbert transform, $r_{E\left[x\left(t\right)\right]}\left(\tau \right)$ is the autocorrelation function of the E[x(t)].

#### 2.3.3. Shock Pulse Index

As the characteristic parameter of vibration signal, kurtosis can reflect the shock characteristics of vibration signals, but the only fly in the ointment is that it is easily influenced by background noise and larger aperiodic impulses. On the other hand, the EHNR, as a new characteristic parameter of vibration signal, can indicate the ratio of harmonic and noise components in vibration signals and that is a good characteristic parameter, but it suffers from interference by other harmonic components such as rotating frequency. Inspired by these two parameters, the SPI that absorbs the advantages and avoids the disadvantages of both indexes was proposed.

According to the dynamic model of the REB [18,29], the vibration signal of the REB usually consists of four parts, i.e., the periodic impulses, the vibration components from related rotating parts, the aperiodic impulses from extraneous sources (large random impulses) and the background noise. The formula as shown:

(26) $$x\left(t\right)=\sum_{i}A_{i}s\left(t-T_{i}\right)+\sum_{j}B_{j}s\left(t-T_{j}\right)+\sum_{k}C_{k}\cos\left(2{\pi f}_{k}t+\varphi_{k}\right)+n\left(t\right),$$

(27) $$s\left(t\right)=e^{-\alpha t}\sin\left(2{\pi f}_{r}t\right),$$

where $A_{i}$ is the amplitude of the ith impulse and $T_{i}$ is the time of its occurrence for the periodic impulses, $B_{j}$ is the amplitude of the jth impulse and $T_{j}$ is the time of its occurrence for the aperiodic impulses, $C_{k}$, $f_{k}$ and $\varphi_{k}$ are the amplitude, frequency and initial phase of the related rotating parts, respectively. n(t) is the background noise. α is the coefficient of resonance damping, $f_{r}$ is the resonance frequency.

**Figure 3:**
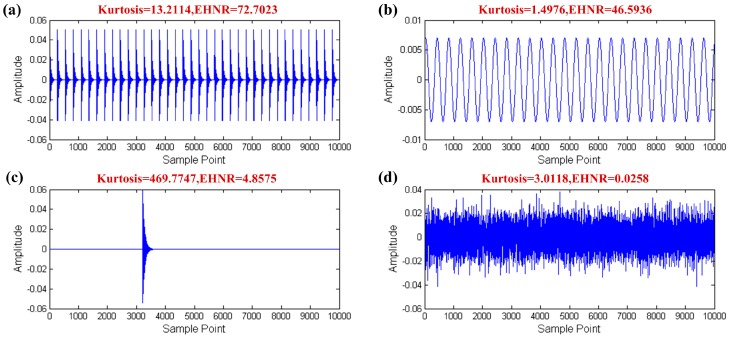
The simulation data of the four parts of the REB vibration signal: (**a**) fault impulses; (**b**) other rotating component; (**c**) aperiodic impulse; (**d**) white noise.

As Figure 3 shows the creation of the simulation signals of the four parts of the REB vibration signal and calculation of the kurtosis and the EHNR values, respectively. The fault impulses with high kurtosis and EHNR value meanwhile, other rotating component only with the high EHNR value, the aperiodic impulse only with the high kurtosis value and where the kurtosis and the EHNR value of the white noise are all not high are shown in Figure 3. Therefore the fault impulses of the REB have two characteristics, i.e., they are impulsive and periodic at the same time and single kurtosis or EHNR index cannot comprehensively summarize their physical characteristics. The organic fusion of the kurtosis and the EHNR, the SPI was put forward as follows:

(28) $$\mathrm{SPI}=\log_{2}\left(1+a*\mathrm{kurtosis}+b*\mathrm{EHNR}\right),$$

where a and b are weight coefficient of the kurtosis and the EHNR, respectively. 0.6 and 0.4 is recommended values of a and b. The kurtosis and the EHNR are all normalized values.

## 3. Properties of the SPI

### 3.1. Capability of Distinguishing Fault Information under High Background Noise

**Figure 4:**
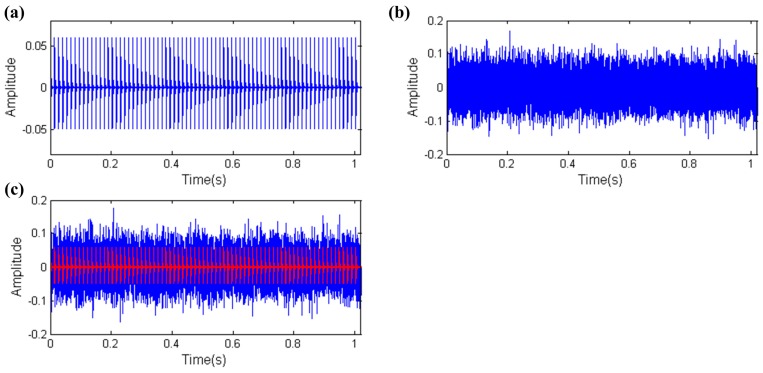
The simulation signal of the REB (including fault impulses and white noise): (**a**) fault impulses; (**b**) white noise; (**c**) mixed signal.

As a part of the test signal, background noise is inevitable and it directly affects the final diagnosis. Therefore it is an important ability of characteristic parameters to distinguish the fault information under high background noise conditions. A simulation is given here to illustrate the capability of the SPI to distinguish the fault information under such high background noise conditions. In this simulation data, the sampling frequency is 20,000 Hz, the number of sampling points is 20,480, the impulse amplitude is 0.06, the coefficient of resonance damping is 620, the resonance frequency is 5800 Hz, the FCF is 74 Hz, and the mean and standard deviation of white noise are 0 and 1, respectively. The fault impulses without other components are shown in Figure 4a. Here, strong white noise as shown in Figure 4b,c indicates the mixed signal of the fault impulses by red line and the white noise. With the aid of the fast-gram technique [12], we use the mixed signal to test the anti-noise ability of three parameters.

**Figure 5:**
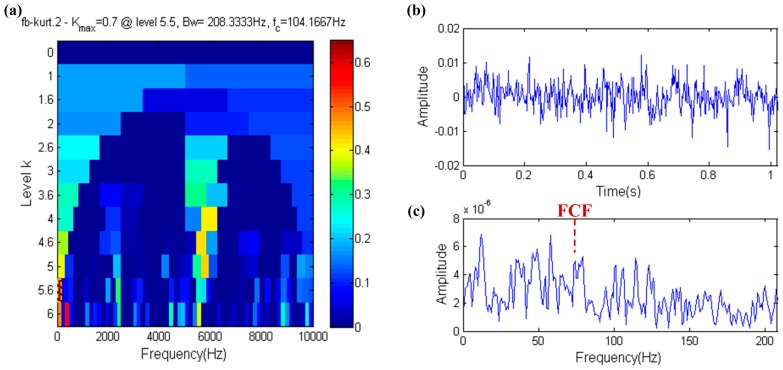
Fast-gram based on the kurtosis: (**a**) Fast kurtogram; (**b**) the filtered signal; (**c**) the envelope spectrum of (**b**).

The fast kurtogram result is shown in Figure 5a. As is seen in this figure, the yellow dotted box is the filter band, which center frequency equals 104.1667 Hz and has a large gap with the resonance frequency (5800 Hz). The filtered signal shown in Figure 5b,c shows the envelope spectrum of the filtered signal. From Figure 5, it is obvious that the fast kurtogram loses effectiveness under strong white noise conditions and the FCF is completely submerged by noise.

**Figure 6:**
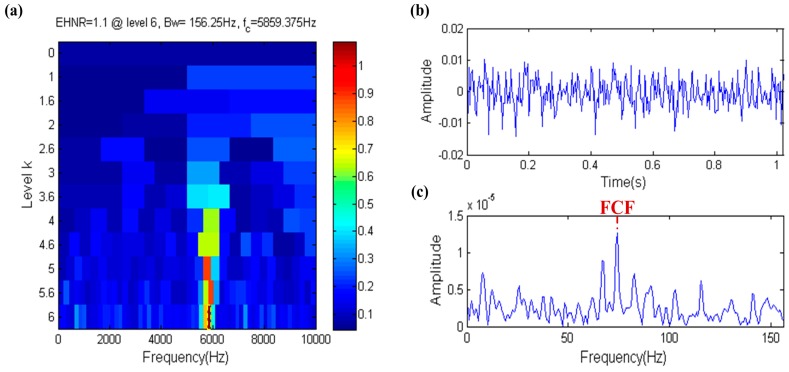
Fast-gram based on the EHNR: (**a**) Fast ehnrgram; (**b**) the filtered signal; (**c**) the envelope spectrum of (**b**).

The result of the fast ehnrgram is shown in Figure 6a, where the filter band in which the center frequency and bandwidth are 5859.375 Hz and 156.25 Hz, respectively, is selected by the fast ehnrgram, which is shown in the yellow dotted box in Figure 6a. The filtered signal and its envelope spectrum are shown in Figure 6b,c, respectively. The FCF can be found in the envelope spectrum of the filtered signal. The center frequency of filter band is nearly equal to the resonance frequency, but the bandwidth of filter band is not big enough. Therefore it shows that the filter band is the optional band but not the optimal band, that is to say that the EHNR has a certain ability to resist noise.

**Figure 7:**
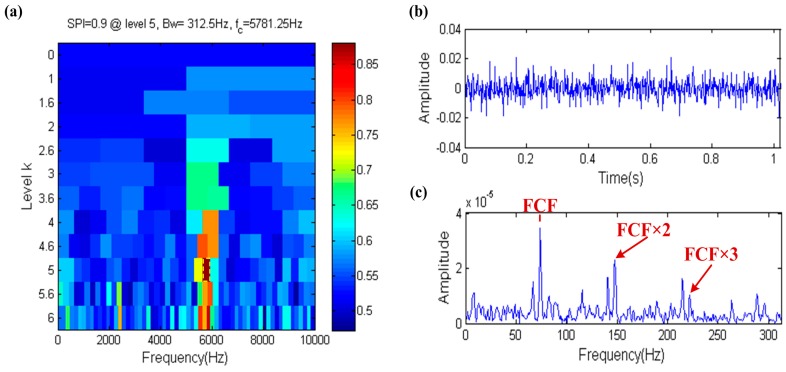
Fast-gram based on the SPI: (**a**) Fast spigram; (**b**) the filtered signal; (**c**) the envelope spectrum of (**b**).

The result of the fast spigram technique is shown in Figure 7, the filter band that the center frequency and bandwidth are 5781.25 Hz and 312.5 Hz, respectively, is selected by the fast spigram, which is shown in the yellow dotted box in Figure 7a. It is observed that the center frequency is close to the resonance frequency and the bandwidth is also big enough, so this means that the fast spigram can accurately identify the fault. The filtered signal is shown in Figure 7b and its waveform has obviously periodic impulses, the envelope spectrum of the filtered signal further proves the effectiveness of fast spigram because the FCF and its high order harmonics are shown clearly in the envelope spectrum, which is shown in Figure 7c. Therefore the SPI has the ability to resist the interference of background noise.

**Figure 8:**
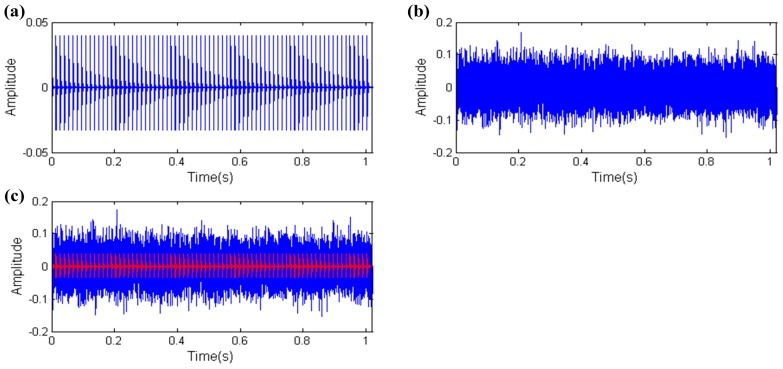
The simulation signal of the REB (including fault impulses and white noise): (**a**) fault impulses; (**b**) white noise; (**c**) mixed signal.

Due to the fact all the collected vibration signals must contain background noise, robustness against background noise is an important ability for characteristic parameters. Therefore, another simulation experiment is designed. In this simulation experiment, the impulse amplitude is set to 0.04 and other parameters are the same with the above simulation experiment to enhance the noise intensity. The simulation signal is shown in Figure 8, the amplitude of the mixed signal is almost triple the fault impulses indicated by the red line in Figure 8c.

**Figure 9:**
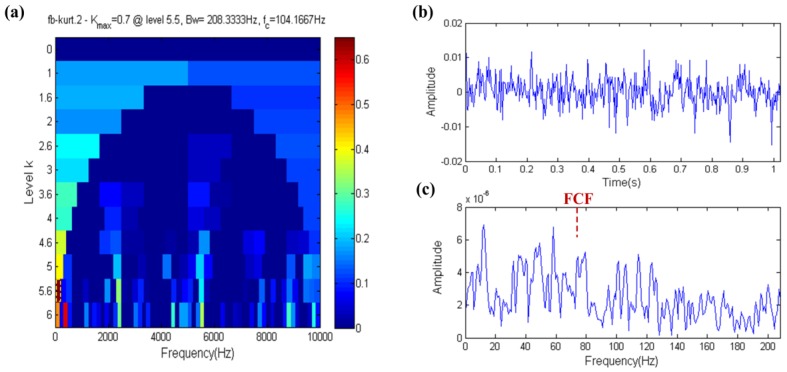
Fast-gram based on the kurtosis: (**a**) fast kurtogram; (**b**) the filtered signal; (**c**) the envelope spectrum of (**b**).

**Figure 10:**
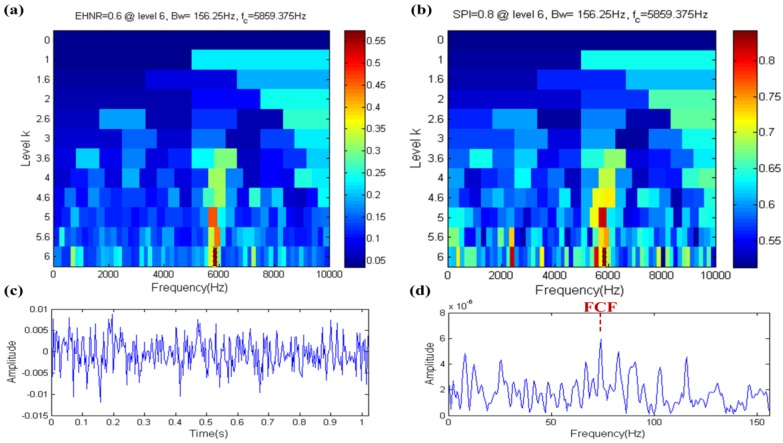
Fast-gram based on the EHNR and the SPI: (**a**) fast ehnrgram; (**b**) fast spigram; (**c**) the filtered signal; (**d**) the envelope spectrum of (**c**).

The fast kurtogram, ehnrgram and spigram results are shown in Figure 9 and Figure 10, respectively. In Figure 9a, the filter band that fast kurtogram selected is obviously not the optimal band because the center frequency of filter band is obviously distinct from the resonance frequency and the envelope spectrum of the filtered signal also proves this conclusion in Figure 9c. In Figure 10a,b the fast ehnrgram and spigram select the same filter band. The center frequency of filter band is close to the resonance frequency so the FCF can be found in Figure 10c. However, the bandwidth of the filter band is also not big enough like in Figure 6a, so the filter band is an optional band but not the optimal band. Although the increase of noise intensity influences the SPI’s ability to identify the fault impulses, the SPI still finds the resonance frequency.

Through the above analysis and discussion, the SPI index is robust in the face of strong background noise and the EHNR index also has this ability, but it not strong enough. The kurtosis index doesn’t have this ability.

### 3.2. Capability of Distinguishing Fault Impulses and Other Harmonic Components

Harmonic components, such as the rotating frequency, are an important part of the vibration signals of rotating machinery. The energy of the rotating frequency of the shaft is usually small in the vibration signal of rotating machinery where the REB is the core component, so the rotating frequency does not affect the extraction of the fault impulses of the REB under normal circumstances. However the energy of the rotating frequency is close to the energy of fault impulses when the REB is in the early failure stage or shaft failure occurs. At this time, identification of the fault impulses of the REB will be affected by the interference of the rotating frequency.

**Figure 11:**
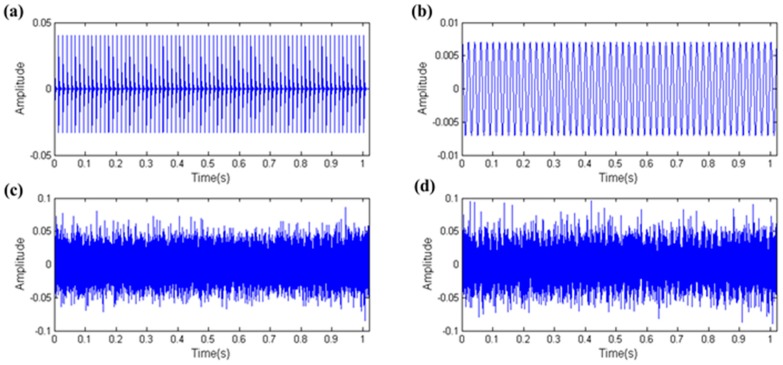
The simulation signal of the REB (including fault impulses, harmonic component and white noise): (**a**) fault impulses; (**b**) harmonic component; (**c**) white noise; (**d**) mixed signal.

On the basis of the simulation signal of Section 3.1 simulation 2, we add a harmonic component that the frequency and amplitude are 50 Hz and 0.007, respectively, and reduce the noise intensity so as to make the harmonic component become a single test index. The corresponding simulation signal is shown in Figure 7. The periodic fault impulses, the harmonic component, the white noise and the mixed signal are shown in Figure 11a–d, respectively. Then we test the capability of distinguishing fault impulses and other harmonic component of these three indexes in accordance with the thinking of Section 3.1.

**Figure 12:**
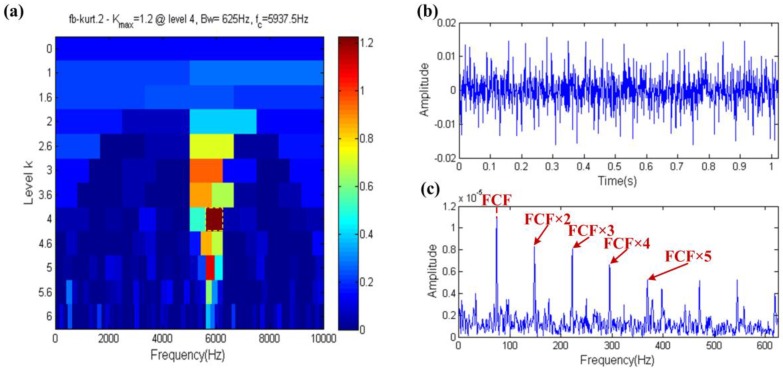
Fast-gram based on the kurtosis: (**a**) Fast kurtogram; (**b**) the filtered signal; (**c**) the envelope spectrum of (**b**).

The result of fast kurtogram technique is shown in Figure 12, where the fast kurtogram selects the filter band that has a center frequency and bandwidth of 5937.5 Hz and 625 Hz, respectively, which is shown in the yellow dotted box in Figure 12a. The center frequency is close to the resonance frequency, so this means that fast kurtogram can accurately identify the fault. The periodic impulses obviously occur in the filtered signal in Figure 12b and its envelope spectrum indicates more clearly harmonics even high order harmonics of the FCF, which is shown in Figure 12c. Hence, the kurtosis index has the ability to resist the interference of harmonic components.

**Figure 13:**
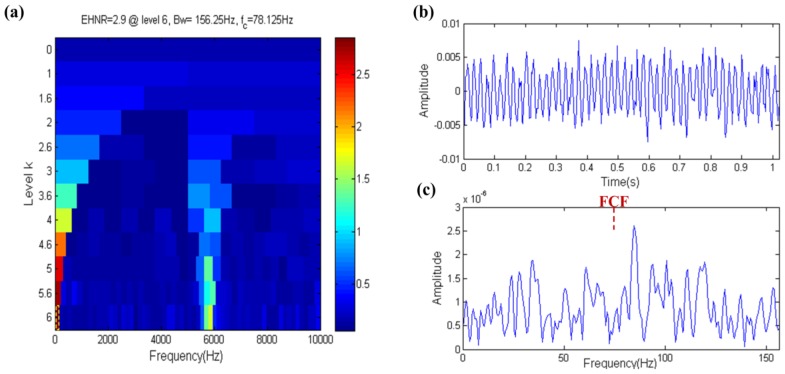
Fast-gram based on the EHNR: (**a**) Fast ehnrgram; (**b**) the filtered signal; (**c**) the envelope spectrum of (**b**).

The result of the fast ehnrgram technique is shown in Figure 13, where the filter band that has a center frequency and bandwidth of 78.125 Hz and 156.25 Hz, respectively, is selected by the fast ehnrgram, which is shown in the yellow dotted box in Figure 13a. There is a large gap between the center frequency and the resonance frequency. This is due to the fact the EHNR index is influenced by the low frequency harmonic component. The waveform of the filtered signal is almost like a harmonic signal in Figure 13b and it is distinct from the fault signal. Moreover the envelope spectrum of the filtered signal cannot indicate the FCF, which is shown in Figure 13c. Therefore the EHNR index is easily influenced by harmonic components.

**Figure 14:**
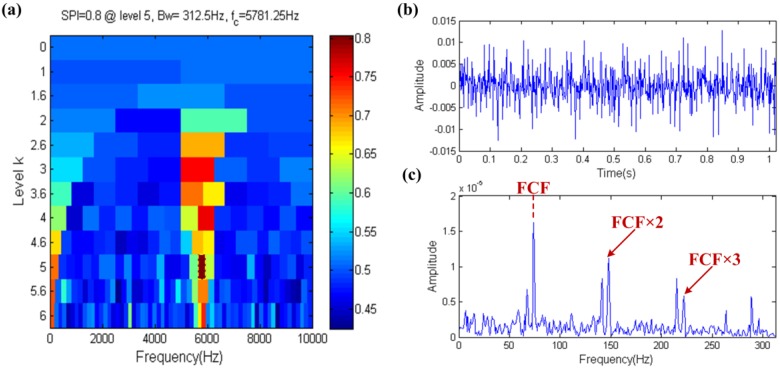
Fast-gram based on the SPI: (**a**) Fast spigram; (**b**) the filtered signal; (**c**) the envelope spectrum of (**b**).

The result of the fast spigram technique is shown in Figure 14, where the filter band with center frequency and bandwidth of 5781.25 Hz and 312.5 Hz, respectively, are selected by the fast spigram, which is shown in the yellow dotted box in Figure 14a. It is observed that the center frequency is close to the resonance frequency, so this means that fast spigram can accurately identify the fault. The filtered signal is shown in Figure 14b and its waveform has obvious periodic impulses, and the envelope spectrum of the filtered signal further proves the effectiveness of the fast spigram because the FCF and its high order harmonics are shown clearly in the envelope spectrum, which is shown in Figure 14c. Therefore the SPI, like kurtosis, also has the ability to resist the interference of harmonic components.

### 3.3. Capability of Distinguishing Fault Impulses and Aperiodic Impulses

Aperiodic impulses, as a random impulse, may be due to random knocks on the test rig or electricity interference and are not common in the vibration signals of the REB, hence they are usually ignored. However once it occurs, an aperiodic impulse can produce a great interference with the diagnosis of the REB, therefore being able to accurately distinguish between aperiodic impulses and the fault impulse is an important feature of the REB fault diagnosis process.

**Figure 15:**
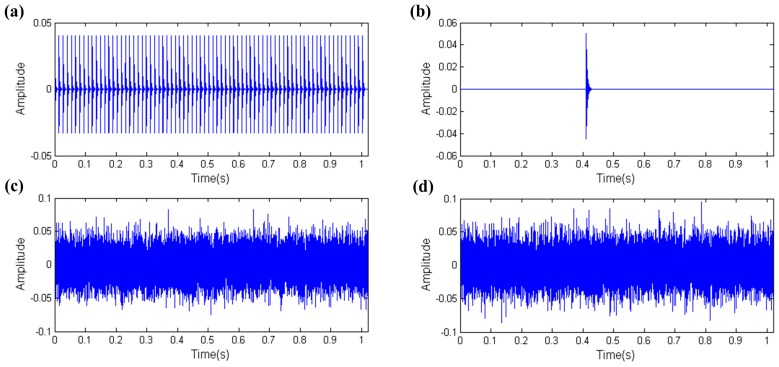
The simulation signal of the REB (including fault impulses, aperiodic impulse and white noise): (**a**) Fault impulses; (**b**) aperiodic impulse; (**c**) white noise; (**d**) mixed signal.

Using the thought of Section 3.1 just like Section 3.2, Figure 15 shows the resulting simulation signal after adding an aperiodic impulse that has a resonance frequency and amplitude of 8700 Hz and 0.05, respectively, in the simulation signal, and moreover reducing the noise intensity so as to make the aperiodic impulse become a single test index. The periodic fault impulses, the aperiodic impulse, the white noise and the mixed signal are shown in Figure 15a–d, respectively.

**Figure 16:**
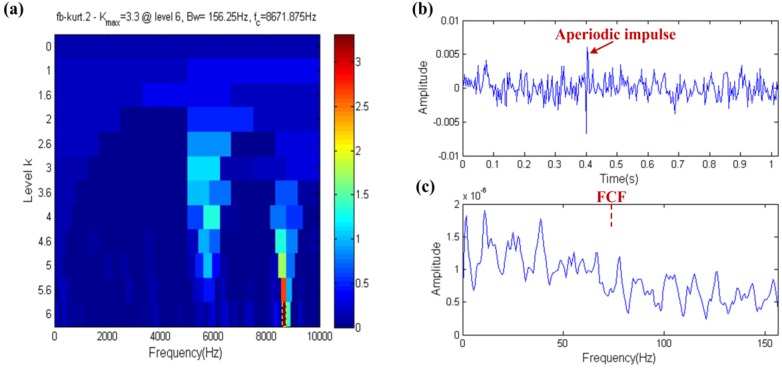
Fast-gram based on the kurtosis: (**a**) Fast kurtogram; (**b**) the filtered signal; (**c**) the envelope spectrum of (**b**).

The fast kurtogram technique result is shown in Figure 16, where the fast kurtogram selects a filter band with a center frequency and bandwidth of 8671.875 Hz and 156.25 Hz, respectively, which is shown in the yellow dotted box in Figure 16a. The center frequency of filter band is close to the resonance frequency of the aperiodic impulse, which means that the diagnosis technology based on kurtosis is easily affected by aperiodic impulses. In Figure 16b, a large aperiodic impulse appears at about 0.4 s and the aperiodic impulse occurs at the same moment in the raw simulation signal, thus it is shown that the filtered signal only manifests the information of the aperiodic impulse and does not show the failure information. The same conclusion can be obtained in Figure 16c, where the FCF cannot be indicated in the envelope spectrum. Therefore, the kurtosis index is vulnerable when it deals with aperiodic impulses.

**Figure 17:**
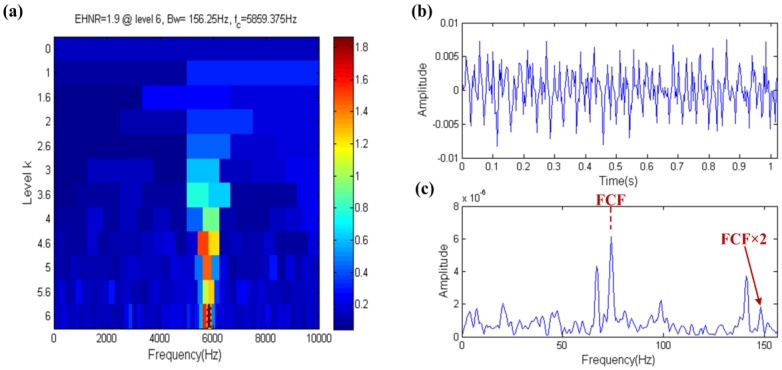
Fast-gram based on the EHNR: (**a**) Fast ehnrgram; (**b**) the filtered signal; (**c**) the envelope spectrum of (**b**).

The result of the fast ehnrgram technique is shown in Figure 17. The filter band of the center frequency and bandwidth of 5859.375 Hz and 156.25 Hz, respectively, is shown in the yellow dotted box in Figure 17a. The center frequency of the filter band is close to the resonance frequency of the fault impulses, that is to say the FCF should be indicated in the envelope spectrum. In Figure 17b, the waveform of the filtered signal has obvious fault impulses and its envelope spectrum also indicates the FCF in Figure 17c. Therefore, the EHNR index is able to eliminate the impact of aperiodic impulses.

**Figure 18:**
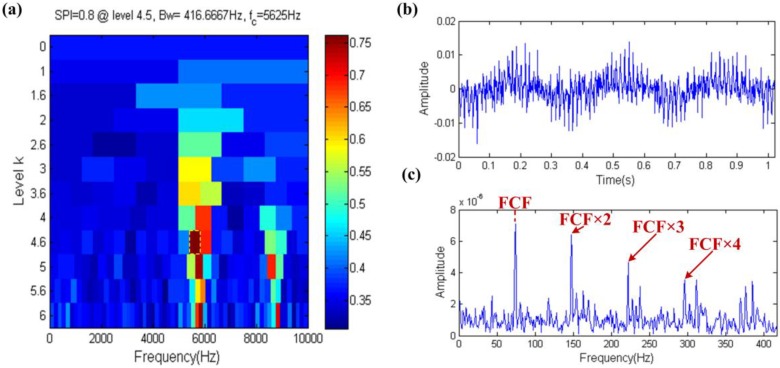
Fast-gram based on the SPI: (**a**) Fast spigram; (**b**) the filtered signal; (**c**) the envelope spectrum of (**b**).

The result of the fast spigram technique is shown in Figure 18, where the filter band with center frequency and bandwidth of 5625 Hz and 416.6667 Hz, respectively, is shown in the yellow dotted box in Figure 18a. The center frequency of filter band is also close to the resonance frequency of the fault impulses just like in Figure 17a, so the same conclusion can be obtained. The filtered signal is shown in Figure 18b and its envelope spectrum obviously shows the FCF even at high order harmonics of the FCF in Figure 18c. Therefore, the SPI index also has the ability to resist the interference of aperiodic impulses.

Through the simulation experiments, the performances of three parameters are tested under the interference of strong noise, harmonic components and aperiodic impulses, respectively. The simulation experiment results shows that the fault diagnosis technology based on the SPI index can be applied to the fault diagnosis of REBs.

## 4. The Proposed Method for Bearing Fault Diagnosis

The fault impulses of the REB cannot only be modulated, but also convoluted with the system transfer function in the transfer process, hence deconvolution is always an important part of the fault diagnosis of the REB. The MCKD, as a powerful deconvolution technique, has been used in some REB fault diagnosis applications and a set of appropriate preset parameters can make it display a better effect. This paper proposed a method using the SPI as the index of the preset parameters optimization of the MCKD and combining this with the TEO demodulation technology. The concrete steps as follows:

Step 1 Load the raw vibration signal measured by the accelerometer and calculate the theoretical fault characteristic frequency $f=\left\{f_{o},f_{e},f_{c},f_{i}\right\}$ by using the geometrical parameters of the REB, then compute the period of impulses T through formula $T=f_{s}/f$. Where $f_{o}$, $f_{e}$, $f_{c}$ and $f_{i}$ are the fault characteristic frequencies of outer race, rolling element, cage and inner race, respectively, $f_{s}$ is the sampling frequency.
Step 2 Set 100 as the lower limit, 500 as the upper limit and 20 as the step size for L; Set 1 as the lower limit, 7 as the upper limit and 1 as the step size for M; then calculate the SPI value of the results of different preset parameters and find out the maximum of the SPI value. Next, use the maximum of the SPI value corresponding to the preset parameters as the MCKD’s optimization parameters.
Step 3 Use the optimization parameters as the preset parameters of the MCKD to filter the raw signal, then obtain the envelope spectrum with the help of the TEO technique.
Step 4 Diagnose the failure type on the basis of the extracted characteristic frequency information from the envelope spectrum.

**Figure 19:**
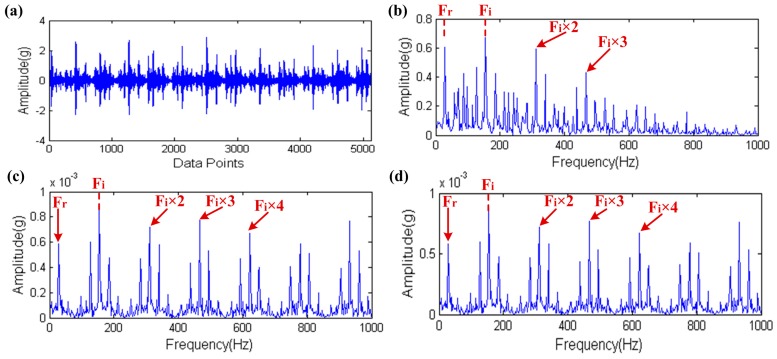
The result of contrast test: (**a**) the raw signal; (**b**) the envelope spectrum of (**a**); (**c**) the envelope spectrum of the filtered signal by the proposed method; (**d**) the envelope spectrum of the filtered signal by the non-simplified method.

**Figure 20:**
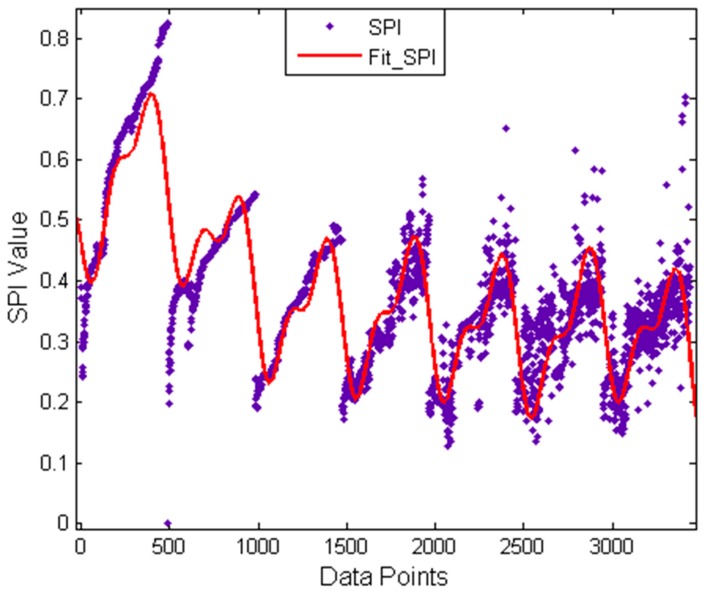
Trend curve of the SPI value.

In Step 2, the proposed method simplifies the preset parameters optimization of the MCKD. This is because a smaller L is not enough to effectively play the role of the MCKD and a smaller step size cannot significantly increase the precision of the solutions but can greatly reduce computational efficiency. In order to demonstrate this point, we design a non-simplified experiment scheme [23]. In this scheme, setting 10 as the lower limit and 1 as the step size for L moreover the upper limit of L and the range of values of M consistent with the proposed method. Then, using a set of inner raceway fault data, which is comes from Case Western Reserve University (CWRU) Bearing Data Center [30] (a detailed description of the experimental procedure is provided in Section 5) as test data to contrast these two methods under the same computational conditions (i3-2120CPU@3.30 GHz). The result of contrast test is shown in Figure 19, where the waveform of the raw signal and its envelope spectrum are shown in Figure 19a,b. The fault impulses are very clearly seen in Figure 19a,b which also proves this point because the inner raceway fault characteristic frequency Fi and its high order harmonic are clearly found. In Figure 19b, the rotating frequency of the shaft is also found and modulation sidebands appear on both sides of the Fi and its high order harmonic, so this may indicate the shaft failure. The envelope spectra of the filtered signal by the proposed method and the non-simplified method are shown in Figure 19c,d. These two envelope spectra are clearer than Figure 19b and the result of the proposed method is very close to the non-simplified method. However, the computation time of the non-simplified method is about 38 times that of the proposed method. Furthermore, the SPI value’s trend of the non-simplified method is shown in Figure 20. The purple dots in the figure are the actual data distribution, while the red line is the fitting curve of the actual data distribution and the type of fit is Sum of Sin Functions. From this figure, the SPI is roughly dispersed in seven areas and each area generally concentrates on a curve. What’s more these seven areas just correspond to seven different M values, that is to say the SPI generally presents the monotone increasing tendency when the M value remains the same and the Fit_SPI curve further proves this conclusion. Therefore it is scientific and effective for the simplification of the proposed method to quickly find the appropriate optimization parameters based on the above two points.

**Figure 21:**
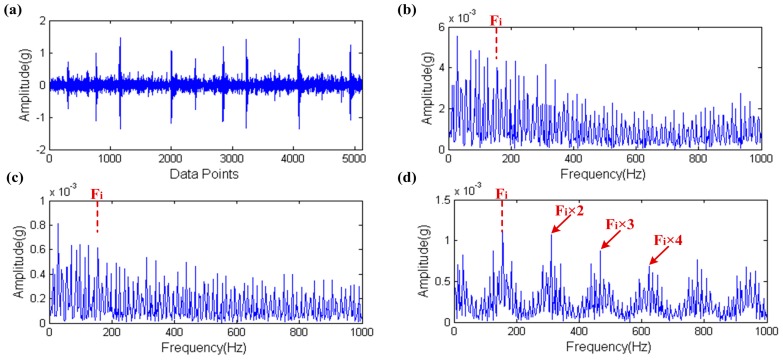
The result of contrast experiment: (**a**) the raw signal; (**b**) the envelope spectrum of the filtered signal by the MED; (**c**) the envelope spectrum of the filtered signal by the original MCKD; (**d**) the envelope spectrum of the filtered signal by the proposed method.

Another inner raceway fault data, which is also comes from the CWRU Bearing Data Center [30] was used to illustrated the strong performance of the proposed method. The proposed method is compared with the MED and the original MCKD by using the same set of data. In this contrast experiment, the filterSize, termIter and termDelta of the MED algorithm are set to 30, 30 and 0.01, respectively, and the filterSize, termIter and M of the original MCKD are set to 0.8T + 0.5, 30 and 1, respectively [20]. The result of this contrast experiment is shown in Figure 21. The waveform of the raw signal is shown in Figure 21a and the fault impulse is very clear. The envelope spectra of the signal filtered by the MED, the original MCKD and the proposed method are shown in Figure 21b–d, respectively. In Figure 21b,c, the inner raceway fault characteristic frequency Fi is almost submerged among the other spectral lines. However, the Fi and its high order harmonic are clearly indicated in Figure 21d, that is to say the proposed method is more powerful than the MED and the original MCKD.

## 5. Experimental Study for Bearing Fault Diagnosis

**Figure 22:**
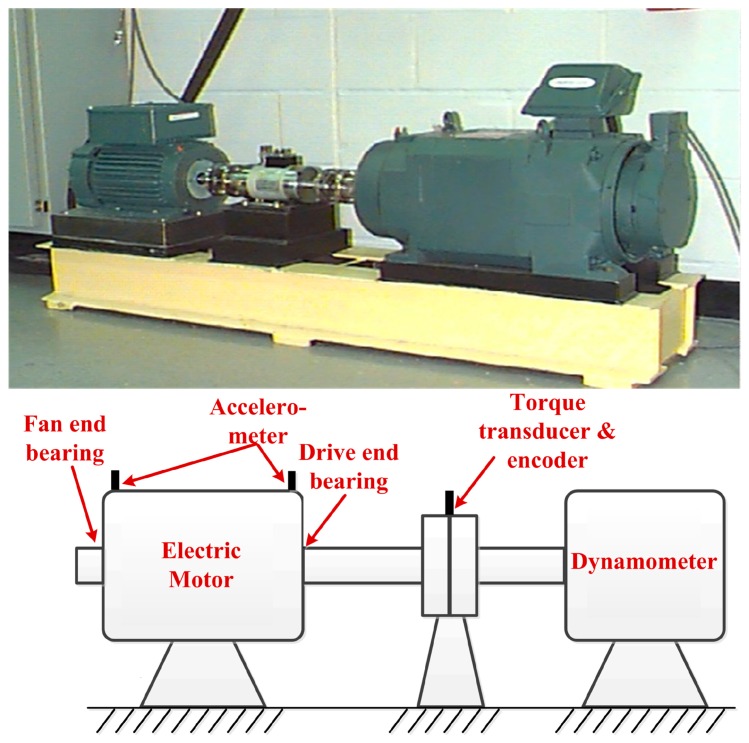
Overview of the test bench and its structure diagram.

The real data comes from Case Western Reserve University (CWRU) Bearing Data Center [30]. The test bench and its structure diagram are shown in Figure 22, the test bench consists of a 2 horsepower motor, a torque transducer and encoder, a dynamometer and control electronics (not shown). Two bearings, identified as the Drive end bearing and Fan end bearing, are installed in both ends of the motor shaft to support the motor shaft itself. The Drive end bearing (6205-2RS JEM SKF) and Fan end bearing (6203-2RS JEM SKF) are both deep groove ball bearings. These two bearings are seeded with faults, which range from 0.007 inches to 0.040 inches in diameter, introduced separately at the inner raceway, rolling element and outer raceway using electro-discharge machining. Faulty bearings are reinstalled into the test motor and vibration data is recorded for motor loads of 0 to 3 horsepower, while the motor speeds ranges from 1796 to 1728 rpm. Accelerometers are attached to the housing with magnetic bases and are placed at the 12 o’clock position at both the Drive end and Fan end of the motor housing. Vibration signals, which are post-processed in a Matlab environment are collected using a 16 channel DAT recorder. Vibration data is collected at 12,000 and 48,000 samples per second for the Drive end bearing, while speed and horsepower data are collected using the torque transducer and encoder and are recorded by hand. In this study, the test data are all collected at 12,000 samples per second for Drive end bearing repetitive but slightly different–consolidate and a random set of testing data is recorded for the inner raceway, rolling element and outer raceway faults, respectively.

### 5.1. Outer Raceway Fault Diagnosis

**Figure 23:**
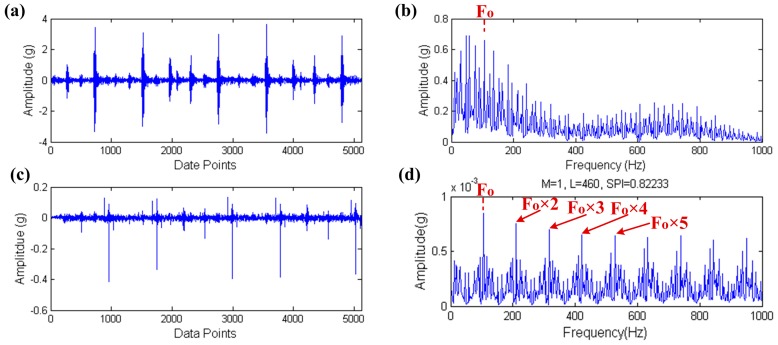
The result of outer raceway fault diagnosis: (**a**) the raw signal; (**b**) the envelope spectrum of (**a**); (**c**) the filtered signal; (**d**) the envelope spectrum of (**c**).

The selected outer raceway fault data is OR021@12_1 and 5120 data points the 50,001th point to the 55,120th point were excerpted in original data in order to improve the computational efficiency. The load is 1 horsepower and opposite to the sensor. What’s more, the motor speed is 1771 rpm, the bearing defect size is 0.021 inches and the theoretical outer raceway fault characteristic frequency is 105.81 Hz. The result of signal processing through the proposed method is shown in Figure 23.

Due to the fact the theoretical fault characteristic frequencies of the REB are all not high (about 100 Hz to 200 Hz), showing the former 1000 Hz frequency band is enough for the envelope spectrum. The whole study uses this thought and what follows need not be repeated. The input signal and its envelope spectrum are shown in Figure 23a,b. Although the outer raceway fault characteristic frequency Fo is found in the envelope spectrum of the input signal, it is not highlighted and other components almost submerge it. Therefore, the envelope spectrum of the input signal cannot clearly indicate the fault. The filtered signal, which is processed by the proposed method is shown in Figure 23c and its envelope spectrum is shown in Figure 23d. Fo is clearly found in the envelope spectrum even the high order harmonics, that is to say bearing faults can be easily found after processing by the proposed method.

### 5.2. Inner Raceway Fault Diagnosis

**Figure 24:**
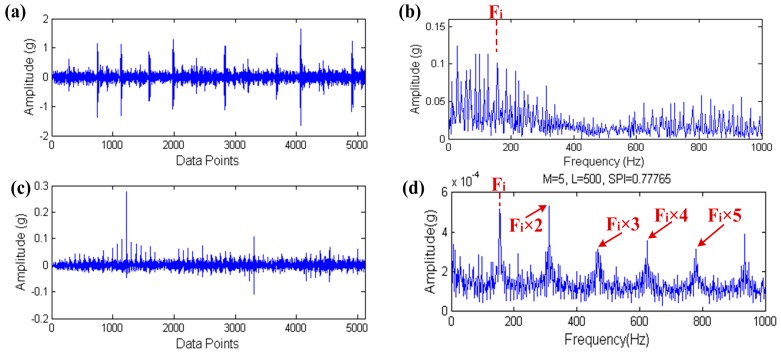
The result of inner raceway fault diagnosis: (**a**) the raw signal; (**b**) the envelope spectrum of (a); (**c**) the filtered signal; (**d**) the envelope spectrum of (**c**).

The selected inner raceway fault data is IR014_3 and we excerpted 5120 data points from the 60,001th point to 65,120th point of the original data in order to improve the computational efficiency. The load is 3 horsepower. What’s more, the motor speed is 1728 rpm, the bearing defect size is 0.014 inches and the theoretical inner raceway fault characteristic frequency is 155.96 Hz. The result of signal processing through the proposed method is shown in Figure 24.

The input signal and its envelope spectrum are shown in Figure 24a,b. The inner raceway fault characteristic frequency Fi is also found in the envelope spectrum of the input signal like Section 5.1, however Fi is not obvious in the multitudinous spectral lines and it is not easy to find it. The filtered signal, which is processed by the proposed method is shown in Figure 24c and its envelope spectrum is shown in Figure 24d. In Figure 24d, Fi and its high order harmonics are clearly highlighted in the envelope spectrum, this means that the inner raceway fault also can be easily found after processing by the proposed method.

### 5.3. Rolling Element Fault Diagnosis

**Figure 25:**
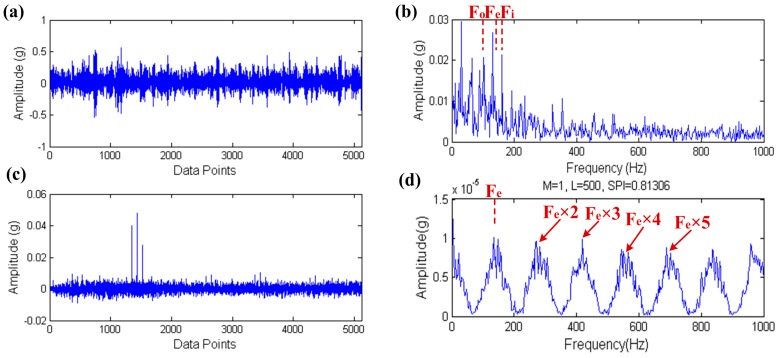
The result of rolling element fault diagnosis: (**a**) the raw signal; (**b**) the envelope spectrum of (a); (**c**) the filtered signal; (**d**) the envelope spectrum of (**c**).

The selected rolling element fault data is B021_0 and we excerpted 5120 data points in the original data from the 70,001th point to the 75,120th point in order to improve the computational efficiency. The load is 0 horsepower. What’s more, the speed of the motor is 1796 rpm, the bearing defect size is 0.021 inches and the theoretical rolling element fault characteristic frequency is 141.09 Hz. The result of signal processing through the proposed method is shown in Figure 25.

The input signal and its envelope spectrum are shown in Figure 25a,b, respectively. In the envelope spectrum of the input signal, the outer and inner raceway fault characteristic frequency Fo and Fi are both found, however the rolling element fault characteristic frequency Fe is submerged by multitudinous spectral lines, and moreover Fo and Fi are both not highlighted because of the interference of other spectral lines. The filtered signal and its envelope spectrum are shown in Figure 25c,d, respectively. Through the processing of the proposed method, Fe and its high order harmonics are obviously visible in Figure 25d.

## 6. Discussion

According to the simulation and real data analysis, the proposed characteristic parameter and method may be enhanced in the future because there are two points worth to be discussed in the process of research.

On the one hand, the number of input data should not be too much when the filter length is up to 500 in the MCDK algorithm. Otherwise the computer will out of memory so the limit of the MCKD limits the application of the proposed method under the requirement of high frequency resolution. 

On the other hand, the SPI index is a characteristic parameter, which obtained from a large number of data study. Therefore, the theory of the periodic impulse’s physical property also needs to improve and the further work should be focus on the improvement of the theory of periodic impulse’ physical property.

## 7. Conclusions

This paper introduces a characteristic parameter named SPI, which can be used in fault diagnosis of REBs. Simulation data is used to test three aspects of the performance of the SPI, i.e., the anti-noise ability, the ability to resist aperiodic impulses and other harmonic components. The performance of the SPI also is compared with kurtosis and the Envelope Harmonic-to-Noise Ratio using simulated data and the conclusions are as follows:

1. Kurtosis index has the ability to resist the interference of the harmonic component, but it is not robust when dealing with the interference of strong noise and aperiodic impulses.
2. The EHNR index can eliminate the influence of strong noise and aperiodic impulses to a certain extent, however the harmonic component will disable it.
3. The SPI index has the ability to identify the fault impulse when facing the interference of strong noise, harmonic components or aperiodic impulses.

All the simulation results show that the SPI, as a dimensionless index, has strong diagnostic ability and it can effectively identify the fault information in the vibration signal, therefore the SPI can be regarded as a kind of characteristic parameter in REB fault diagnosis. Then, we proposed a fault diagnosis method where the SPI is applied to the parameter optimization of the MCKD for a set of real vibration data. Through an experimental study of real vibration signals, the SPI shows a strong ability to identify fault information in the parameter optimization of the MCKD and the experimental data results indicate clearly the FCF and its high order harmonics, that is to say SPI is feasible as an optimization index. Moreover, according to the experimental study performed so far, it can be concluded that the proposed method is more powerful than the MED and the original MCKD under the same conditions, as the MCKD has a strong deconvolution ability under the conditions of appropriate preset parameters in the REB fault diagnosis, and the Teager energy operator has a strong signal demodulation performance. On basis of its strong performance, the Teager energy operator has a brilliant future in demodulation of the field of mechanical fault diagnosis.
